# Fascin Drives Breast Cancer Cell Proliferation Partly by Modulating the Cell Cycle Checkpoint Regulators of the G1-S Phase

**DOI:** 10.3390/cells14231839

**Published:** 2025-11-21

**Authors:** Hazem Ghebeh, Huda K. Al-Nasrallah, Marwa Elfoly, Alanoud Aldossry, Asma Tulbah, Taher Al-Tweigeri, Monther Al-Alwan

**Affiliations:** 1 Innovation and Research, King Faisal Specialist Hospital and Research Centre, MBC:03-99, P.O. Box 3354, Riyadh 11211, Saudi Arabia; hghebeh@kfshrc.edu.sa (H.G.); halnasrallah@kfshrc.edu.sa (H.K.A.-N.); malfoly@kfshrc.edu.sa (M.E.); aaldossry@kfshrc.edu.sa (A.A.); 2 College of Medicine, Al-Faisal University, Riyadh 11533, Saudi Arabia; 3 Department of Botany and Microbiology, College of Science, King Saud University, Riyadh 11451, Saudi Arabia; 4 Department of Pathology and Laboratory Medicine, King Faisal Specialist Hospital and Research Centre, Riyadh 11211, Saudi Arabia; tulbah@kfshrc.edu.sa; 5 Cancer Center of Excellence, King Faisal Specialist Hospital and Research Centre, Riyadh 11211, Saudi Arabia; ttwegieri@kfshrc.edu.sa

**Keywords:** breast cancer, fascin, proliferation, cell cycle

## Abstract

Breast cancer (BC) is the most frequently diagnosed malignancy in women worldwide. Despite therapeutic advances, disease relapse and metastasis remain major challenges and drivers of mortality. Fascin, an actin-bundling protein, promotes BC progression by enhancing drug resistance. However, the role of fascin in proliferation, a hallmark of cancer, and the underlying mechanism remain poorly elucidated. In this study, bioinformatics analysis of publicly available BC datasets, gene manipulation (gain and loss of function) in BC cell lines, flow cytometry, Western blots, and a real-time cell analyzer (RTCA) were employed to assess the role of fascin in proliferation. The clinical relevance of bioinformatics data and in vitro findings was assessed in BC patient samples using immunohistochemistry. *FSCN1* expression exhibited a significant correlation with proliferation signature scores in BC datasets. Ectopic expression of fascin in fascin-negative SK-BR-3 and its silencing in fascin-positive MDA-MB-231 BC cell lines demonstrated its direct role in driving proliferation. In-depth bioinformatics analyses revealed a significant correlation between *FSCN1* and the cell cycle signature score, particularly the G1-S signature score gene set. Indeed, fascin accelerated the cell cycle progression of synchronized cells from the G to S phase. Mechanistically, fascin upregulated nuclear SKP2 levels and reduced p27 expression—important G1-S cell cycle checkpoint regulators. Immunohistochemistry in samples from 68 patients demonstrated significant correlations between fascin and Ki-67 expression, in addition to SKP2 overexpression and p27 downregulation. Collectively, these data demonstrate the role of fascin as a driver of the G1-S-phase transition during cell cycle proliferation, thereby revealing new opportunities for targeted therapeutic intervention.

## 1. Introduction

Breast cancer (BC) is the most prevalent malignancy in women worldwide. Identifying biomarkers that drive BC aggressiveness could consequently lead to their therapeutic targeting, ultimately resulting in more effective treatment outcomes. Fascin is an actin-bundling protein that has restricted expression in normal cells and regulates many cellular processes, including cell shape and motility [1]. It is aberrantly expressed in many transformed epithelial cells, including BC (reviewed in [2]). In cancer, fascin expression has been reported to regulate many signaling pathways that support aggressive cancer cell behavior and promote disease progression. For example, fascin suppressed BC metastasis suppressor 1 (BRMS1) while it enhanced the NF-κB signaling pathway to promote BC migration and invasion. Importantly, fascin correlated with disease relapse and metastasis [3]. Subsequently, we showed that fascin enhances the focal adhesion kinase (FAK) and AKT signaling pathways in BC to confer resistance to chemotherapy, and it correlates with poor survival outcomes [4]. Moreover, we showed that fascin positively regulates β1 integrin expression in BC and their co-expression promotes aggressive cell behavior in vitro and poor survival outcome in patients [5]. We also showed that fascin activates FAK through phosphorylation to trigger the β catenin signaling pathway and promote the aggressive behavior of BC in vitro, which correlates with worse clinical outcomes in patients [6]. Liu, H et al. have elegantly reviewed many of the signaling pathways and downstream targets that are regulated by fascin (reviewed in [2]).

The relationship between fascin and cancer cell proliferation has not been thoroughly studied, with somewhat inconsistent results. Despite the scarcity of studies analyzing the relationship between fascin and proliferation, only a few of them investigated the underlying mechanism of action, mainly in esophageal squamous cell carcinoma [7], non-small cell lung cancer [8,9], and melanoma [10]. Given the effect of cell proliferation on tumor growth, there is a need to understand the relationship between fascin and proliferation in cancer cells and to delineate the underlying mechanisms.

In this study, we demonstrate that fascin expression in BC enhances proliferation and accelerates cell cycle progression through G1-S cell cycle restriction, SKP2 upregulation, and p27 suppression. Immunohistochemistry in BC patient samples further revealed a significant correlation between fascin expression and higher levels of the standard proliferation marker (Ki-67) and nuclear SKP2, alongside reduced nuclear p27. Elucidating the mechanisms through which fascin contributes to proliferation and tumor growth may help in developing more effective strategies for targeting this pathway in clinical settings.

## 2. Materials and Methods

### 2.1. Cells and Reagents

The human BC cell lines SK-BR-3 (HTB-30) and MDA-MB-231 (HTB-26) were procured from ATCC (Manassas, VA, USA). The cells were maintained in complete RPMI-1640 (for SK-BR-3) and DMEM (for MDA-MB-231) containing 10% FBS, 200 mM L-glutamine, and an antibiotic–antimycotic solution (Invitrogen; Carlsbad, CA, USA) at 37 °C in a 5% CO_2_ humidified atmosphere.

An anti-human fascin antibody (Agilent DAKO; Santa Clara, CA, USA) and a secondary APC-labeled goat anti-mouse IgG1 antibody (Jackson ImmunoResearch Labs; West Grove, PA, USA) were used for fascin detection via flow cytometry. The anti-fascin (D1A8), anti-SKP2 (D3G5), anti-p27 (D69C12), and anti-p21 (12D1) antibodies from Cell Signaling Technology (Danvers, Massachusetts, VA, USA) were used for protein detection via Western blot. The anti-Lamin B and anti-Ki-67 antibodies were from Abcam (Cambridge, UK), while the anti-GAPDH antibody was from Santa Cruz Biotechnology (Dallas, TX, USA).

### 2.2. Fascin Induction

SK-BR-3 BC cells are inherently fascin-negative. Empty ORF (control; LP-NEG-Lv105-020002) or fascin ORF (LP-D0369-Lv105-0205-S) lentiviral particles (Genecopoeia; Rockville, MD, USA) were used to generate fascin-negative and fascin-positive cells, respectively, as previously described [5]. Post-transfection selection and cloning were used to generate fascin-positive SK-BR-3 (SOF) cells and a fascin-negative (SON) control cell. Fascin knockdown in the inherently fascin-positive MDA-MB-231 BC cells was achieved using a scrambled or fascin-specific ShRNA to generate fascin-positive (ShCon) and fascin-negative (ShFSCN) cells, as previously described [3]. The confirmation of fascin expression or knockdown was routinely checked at the RNA and protein levels.

### 2.3. RT-qPCR

Total RNA and cDNA were prepared from cells, and quantitative Real-Time PCR (RT-qPCR) was carried out as previously described [3]. RT-qPCR was run using gene-specific TaqMan fluorogenic probes (Applied Biosystems, Thermo Fisher Scientific, Waltham, MA, USA) on the Applied Biosystems 7500 Fast detection system. The results were presented as the mean of triplicates ± SD from three independent experiments.

### 2.4. Flow Cytometry

Fascin was stained as previously described [3]. Samples were acquired (10,000 events) and analyzed via flow cytometry (LSR II; Becton Dickinson, Mountain View, CA, USA).

Cell cycle analysis was performed using flow cytometry as previously described [11]. Briefly, SK-BR-3 cells (60–80 confluent) were washed with PBS and serum-starved for 10 h, followed by 16 h of incubation with 4 μg/mL of aphidicolin to synchronize the cells in the G1/S phase. The cells were then sub-cultured in complete media and harvested at the indicated time points into FACS tubes. After washing, cells were fixed in 70% ethanol, stained with propidium iodide, and acquired on the LSR II flow cytometer.

### 2.5. RTCA

Cells were seeded at 22,000 per well in CIM-plate 16, and the xCELLigence Real-Time Cell Analysis (RTCA) instrument (ACEA Biosciences Inc., San Diego, CA, USA) was used to monitor cell proliferation over 72 h, as previously described [11]. The xCELLigence software (RTCA software 2.0.0.1301) was used to acquire data and calculate the average proliferation of all replicates via increases in the slope (1/h) of the curve over 72 h. The data were presented as the mean of triplicates ± SD from three independent experiments.

### 2.6. Quantitative Immunofluorescence

To assess protein expression using the immunofluorescence technique, cells were cytospun on slides, fixed, and stained as previously described [11]. Images were captured on a BD Pathway 855 Image analyzer, and the mean fluorescence intensity (MFI) was calculated using BD AttoVision image acquisition software version 1.5 (BD Biosciences; San Jose, CA, USA) as previously described [11].

### 2.7. Western Blot

After collecting cell pellets from different groups, cell lysates were run on a gel and transferred to membranes as previously described [11]. The desired primary antibody was then added to the membrane overnight, followed by incubation with an HRP-conjugated secondary antibody for 45 min. The bound antibodies were detected using the chemiluminescence Super Signal System (Thermo Fisher Scientific, Grand Island, NY, USA) and captured with an ImageQuant LAS4010 Biomolecular Imager (GE Healthcare, Pittsburgh, PA, USA). Imager bands were quantified and analyzed using ImageJ software version 1.54r.

### 2.8. Expression Analysis of Human Breast Tumor Microarray Datasets

Bioinformatics analyses of invasive breast carcinoma were conducted using the publicly available METABRIC dataset (TCGA, Nature 2012 and Nature Communications 2016), which includes 1980 samples reported in the original publication by Curtis et al. (Nature, 2012). Data were downloaded and analyzed using R software version 4.4.2. The association between *FSCN1* expression and proliferation was assessed using several proliferation-related gene sets, including the following: the proliferation signature (MSigDB, GO:0008283; C5:GO Biological Process, 512 genes), the cell cycle signature (MSigDB, GO:0007049; C5:GO Biological Process, 1705 genes), the G1–S signature (MSigDB, GO:0000082; C5:GO Biological Process, 27 genes), and the G2M signature (MSigDB, HALLMARK_G2M_CHECKPOINT; source publication: Pubmed 26771021; 200 genes). Gene expression data were first normalized to z-scores on a per-gene basis across the entire patient cohort to ensure standardization. Gene signature scores were calculated as the average expression value of all genes within each signature for every patient sample. Patients were further stratified into high- and low-expression groups based on the median gene value. Pearson’s correlation coefficient (R) and all related statistical analyses were performed as previously described [11].

### 2.9. Immunohistochemistry

Formalin-fixed paraffin-embedded (FFPE) tissue sections from 68 BC patients were prepared and processed as previously described [5]. All stainings were performed and assessed by an anatomical pathologist, scoring SKP2, p27, p21, Ki-67, and PD-L1 as previously described [11]. The anti-fascin (55K-2) antibody was from DAKO (Santa Clara, CA, USA), and anti-p21 (12D1) and anti-SKP2 (D3G5) were from cell signaling technology (Danvers, MA, USA), while anti-p27 (F8) was from Santa Cruz Biotechnology (Dallas, TX, USA), anti-Ki-67 (MIB-1) was from Agilent DAKO (Santa Clara, CA, USA), and anti-PD-L1 (SP263) was from Ventana (Oro Valley, AZ, USA). We applied previously established cutoff values: 10% for SKP2 and 50% for p27, as reported by the authors of [12], and 20% for Ki-67, as described by the authors of [13]. 

## 3. Results

### 3.1. Bioinformatics Analysis Reveals a Correlation Between FSCN1 Expression and Proliferation Markers in BC

**Figure 1:**
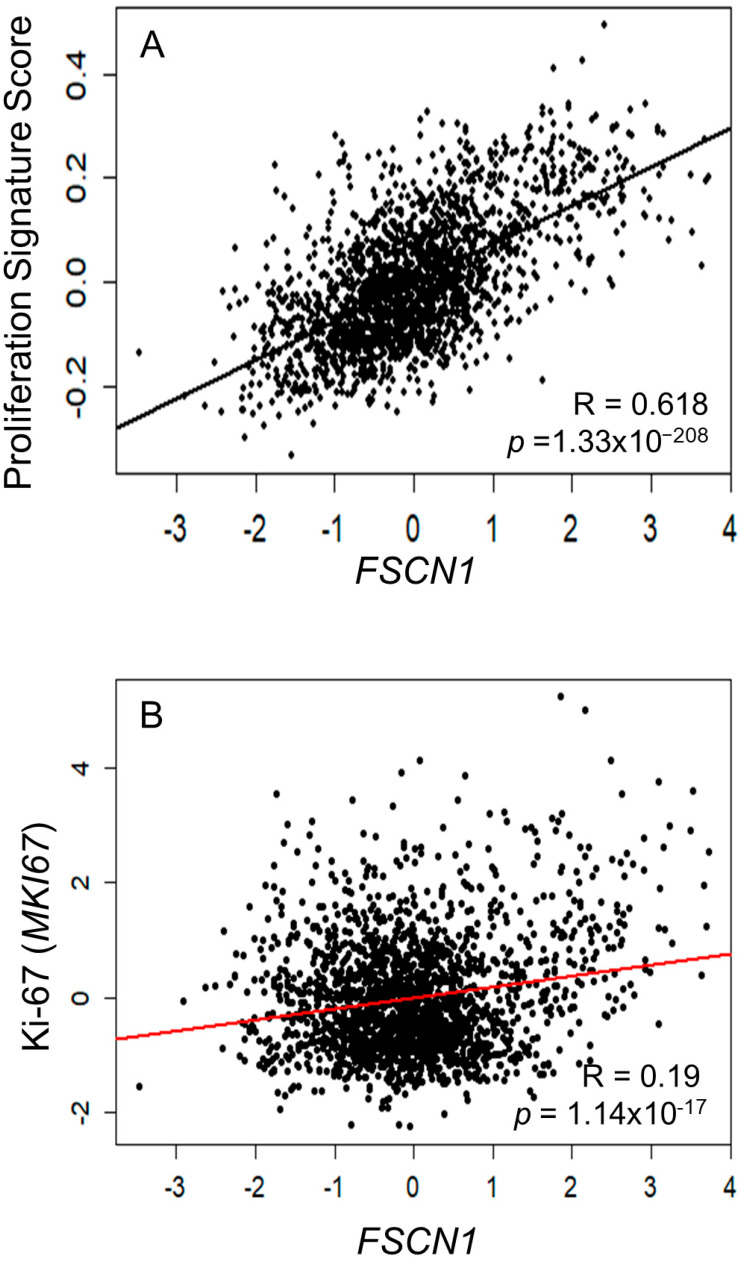
(**A**,**B**): Fascin expression in BC patients correlates with proliferation signature score. Scatter plot showing the correlation between *FSCN1* mRNA expression and 514 gene proliferation signature score (**A**) or Ki-67 (encoded by MKI67) (**B**) using the METABRIC BC dataset (*n* = 1980). Pearson’s correlation coefficients (R) and corresponding *p*-values are displayed on the plot.

We previously reported a significant correlation between fascin expression and larger tumor sizes in BC patients [3]. To examine whether this association reflects a fascin-mediated effect on tumor cell proliferation, we analyzed publicly available TCGA BC microarray datasets to assess the relationship between *FSCN1* mRNA levels and the proliferation signature score, which is composed of 514 proliferation-related genes. Figure 1A shows a strong correlation between *FSCN1* and the proliferation signature score (R = 0.618, *p* = 1.33 × 10^−208^). Specifically, there was a statistically significant, albeit weak, correlation between *FSCN1* and Ki-67 (encoded by *MKI67*; R = 0.19, *p* = 1.14 × 10^−17^), a global proliferation marker that correlates with faster tumor growth (Figure 1B). Altogether, bioinformatics analysis revealed a significant correlation between fascin and markers of proliferation.

### 3.2. Fascin Expression Drives the Proliferation of BC Cells In Vitro

**Figure 2:**
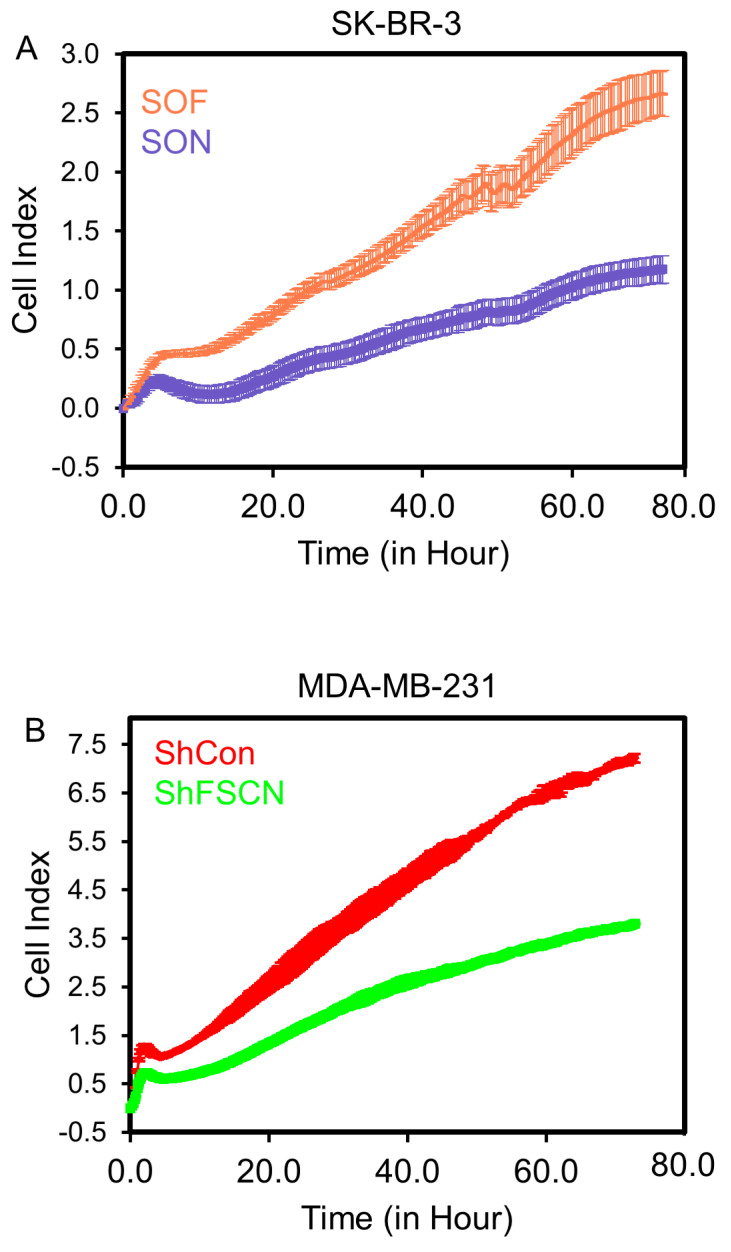
(**A**,**B**): Fascin expression promotes BC cell proliferation and cell cycle progression. (**A**) RTCA showing enhanced proliferation of SOF compared to SON (**A**) and ShCon compared to ShFSCN (**B**). The results (mean ± SD) are representative of 5 (SK-BR-3) and 3 (MDA-MB-231) independent experiments.

To investigate whether fascin directly drives proliferation, fascin ORF was introduced into the inherently fascin-negative SK-BR-3 BC cell line to generate fascin-expressing cells (SOF). Fascin expression in SOF was confirmed at the RNA level via RT-qPCR (Supplementary Figure S1A), and at the protein level, it was confirmed via flow cytometry (Supplementary Figure S1B). Real-time cell proliferation monitoring using RTCA demonstrated a significantly increased rate of proliferation in fascin-expressing SK-BR-3 (SOF) cells compared to empty ORF control (SON) cells (Figure 2A). To validate its role in BC cell proliferation, fascin was silenced (using ShRNA) in MDA-MB-231 cells (Supplementary Figure S2), another BC cell line that is inherently fascin-positive. Fascin knockdown (ShFSCN) in MDA-MB-231 cells resulted in reduced proliferation compared to their fascin-expressing (ShCon) counterparts (Figure 2B). Collectively, fascin expression in SK-BR-3 (SOF vs. SON) and MDA-MB-231 (ShCon vs. ShFSCN) cells supports a direct role for fascin in promoting BC cell proliferation.

### 3.3. FSCN1 Expression in BC Patient Samples Correlates with G1-S Signature Score

**Figure 3:**
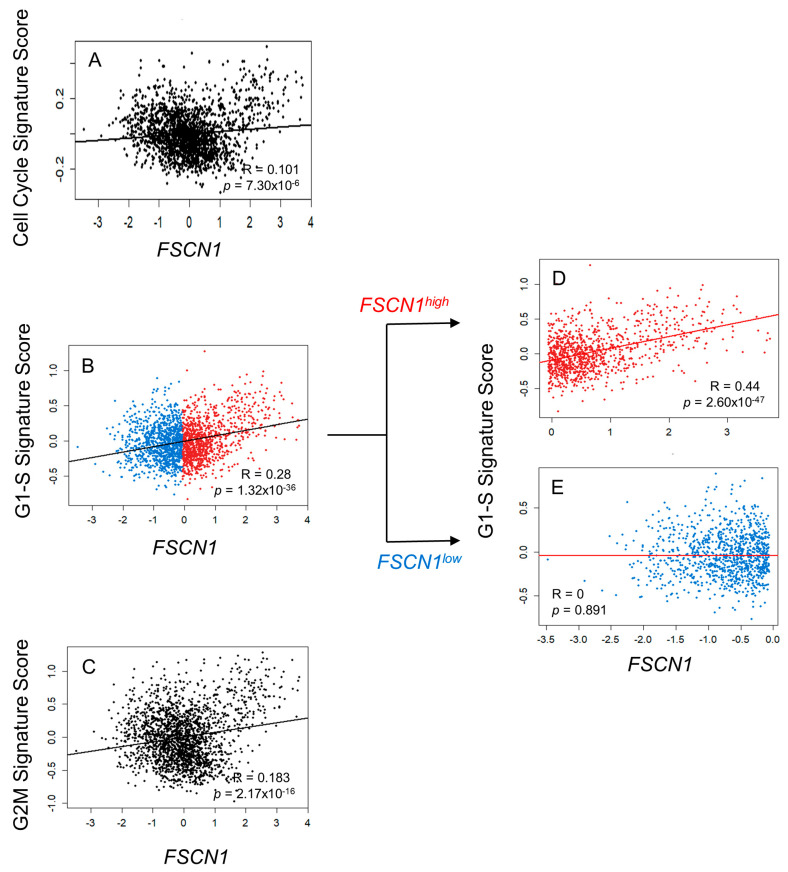
(**A**–**E**): Fascin expression in BC patient samples correlated with the G1-S proliferation signature score. Scatter plot showing the correlation between *FSCN1* expression and the 1705 cell cycle signature score (**A**), the 27 G1-S gene signature score (**B**), and the 200 G2M gene signature score (**C**) using the METABRIC BC dataset (*n* = 1980). Patients were stratified into (**D**) *FSCN1*^high^ (*n* = 990) and (**E**) *FSCN1*^low^ (*n* = 990) groups based on the median *FSCN1* expression level. Scatter plots illustrate the correlation between *FSCN1* expression and the G1-S signature score within each subgroup. Pearson’s correlation coefficients (R) and corresponding *p*-values are displayed on each plot.

**Figure 4:**
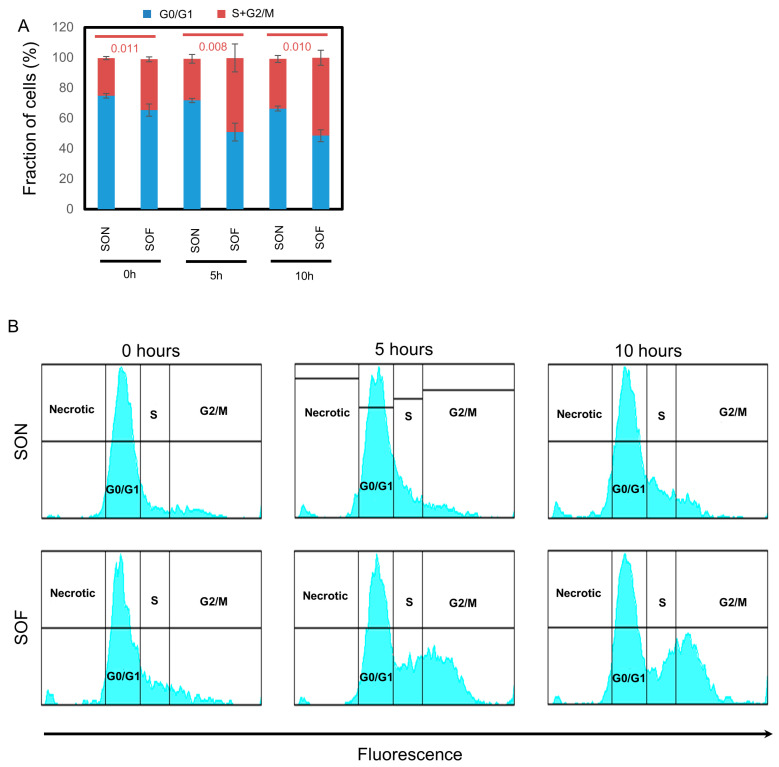
(**A**,**B**): Fascin expression promotes BC cell cycle progression. Bar graph of cell cycle analysis of SK-BR-3 (**A**) showing fractions (percentages) of cells after drug (aphidicolin) washing and complete medium supplementation. The results (mean ± SD) are representative of 3 independent experiments, showing the percentage of cells in the G0/G1 (cycling) or S + G2/M phases. (**B**) Representative flow cytometry cell cycle analysis.

To investigate how fascin promotes proliferation, we focused on its relationship with genes related to the cell cycle. We used the METABRIC BC publicly available dataset to assess *FSCN1* expression in relation to the cell cycle signature score (1705 genes). *FSCN1* showed a statistically significant, albeit weak, correlation with the cell cycle signature score (R = 0.101, *p* = 7.30 × 10^−6^; Figure 3A), indicating a relationship between fascin and the cell cycle. Further analysis of the correlation of *FSCN1* with different phases of the cell cycle showed a statistically significant correlation between fascin with the G1-S signature score (27 genes) (r = 0.28, *p* = 1.32 × 10^−36^; Figure 3B) and with the G2M signature score (200 genes) (r = 0.185, *p* = 2.17 × 10^−16^; Figure 3C). As the *FSCN1* correlation with the G1-S signature score was higher and more significant, we then focused on the G1-S phases of the cell cycle. Stratified analysis revealed that its significant correlation with the G1-S signature score became more evident in the *FSCN1*^high^ group (r = 0.44, *p* = 2.60 × 10^−47^; Figure 3D), whereas it was completely lost in the *FSCN1*^low^ group (r = 0, *p* = 0.89; Figure 3E), hinting at the role of fascin overexpression in promoting cell proliferation via an effect on the G1-S transition phase of the cell cycle. Therefore, we examined the impact of fascin expression on the G1-to-S phase transition of the cell cycle by synchronizing BC cells in the G1-S cell cycle restriction point using aphidicolin. Although the SOF group initially exhibited a slightly higher fraction of S + G2/M phase cells at the 0 h time point, likely due to a small subset of cells not being fully arrested at the G1–S restriction point, this group demonstrated significantly greater fold increases at both 5 and 10 h after drug removal (Figure 4A,B). Compared to their respective 0 h values, SOF cells maintained a significantly higher S + G2/M fraction at 5 h (1.45-fold; *p* = 0.032) and 10 h (2.06-fold; *p* = 0.026). In contrast, the SON group showed a significant increase in the S + G2/M fraction only at 10 h (1.31-fold; *p* = 0.025) but not at 5 h (1.10-fold; *p* = 0.183), indicating slower G1-S entry and overall delayed cell cycle progression. These findings demonstrate that fascin drives BC proliferation by regulating genes that promote the cell cycle transition from the G1 to S phase.

### 3.4. FSCN1 Modulates the Expression of SKP2-p27 Cell Cycle Checkpoint Regulators

**Figure 5:**
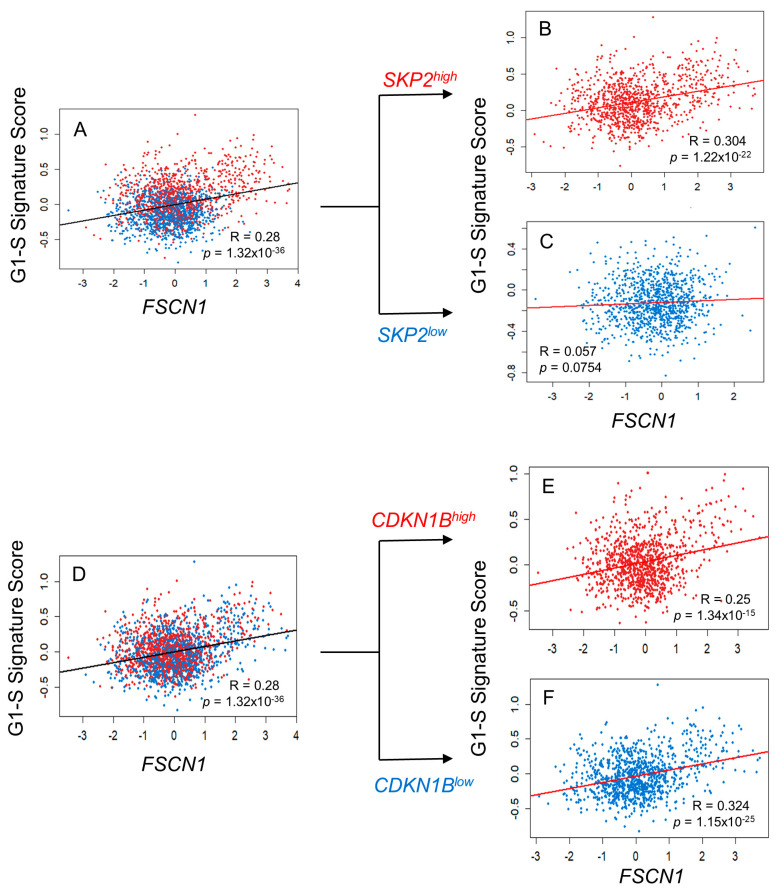
(**A**–**F**): Fascin correlation with the G1-S proliferation signature score is SKP2-dependent. Scatter plot showing the correlation between *FSCN1* expression and 27-G1-S gene signature score (**A**,**D**) using the METABRIC BC dataset (*n* = 1980). Patients were stratified into (**B**) *SKP2*^high^ (*n* = 990) and (**C**) *SKP2*^low^ (*n* = 990) groups based on the median *SKP2* expression level. Patients were stratified into (**E**) *CDKN1B*^high^ (*n* = 990) and (**F**) *CDKN1B*^low^ (*n* = 990) groups based on the median *CDKN1B* expression level. Scatter plots illustrate the correlation between fascin expression and the G1-S signature score within each subgroup. Pearson’s correlation coefficients (r) and corresponding *p*-values are displayed on each plot.

We recently reported that the immune checkpoint PD-L1 promotes BC proliferation by facilitating cell cycle entry through the SKP2–p27/p21 axis [11], which is part of the G1-S signature. Given our previous finding of a significant correlation between fascin and PD-L1 (*p* = 0.008) [3], we investigated whether fascin promotes BC cell proliferation through the same regulatory axis. To this end, we analyzed the publicly available METABRIC BC dataset to examine the association between *FSCN1* mRNA levels and those of *SKP2*, p27 (encoded by *CDKN1B*), or p21 (encoded by *CDKN1A*). Bioinformatics analysis showed a significant correlation between *FSCN1* and *SKP2* (r = 0.36, *p* = 1.97 × 10^−60^; Supplementary Figure S3). While *FSCN1* exhibited a statistically significant but weak inverse correlation with p27 (r = −0.07, *p* = 0.0032; data not shown), it also exhibited a statistically significant, albeit weak, positive correlation with p21 (r = 0.06, *p* = 0.0095; data not shown). To further examine the relationship between *FSCN1* and G1-S signature scores and *SKP2*, cases were stratified into *SKP2*^high^ and *SKP2*^low^. Interestingly, the *FSCN1* correlation with the G1-S signature score was stronger in *SKP2*^high^ (R = 0.302, *p* = 1.22 × 10^−22^), while this association was lost in the *SKP2*^low^ (R = 0.057, *p* = 0.0754) group (Figure 5A–C). Furthermore, *FSCN1*’s correlation with the G1-S signature score was lower in the *CDKN1B*^high^ group (R = 0.25, *p* = 1.34 × 10^−15^) than in *CDKN1B*^low^ (R = 0.324, *p* = 1.15 × 10^−25^) group (Figure 5D–F). Similarly, *FSCN1* exhibited a weak but statistically significant positive correlation with the cell cycle signature score in *SKP2*^high^ (R = 0.206, *p* = 5.46 × 10^−11^) and *CDKN1B*^low^ (R = 0.168, *p* = 1.65 × 10^−7^) as opposed to a significant inverse correlation in the *SKP2*^low^ (R = −0.358, *p* = 2.27 × 10^−31^) and no significant difference in the *CDKN1B*^high^ (R = 0.039, *p* = 0.219) groups (Supplementary Figure S4). Collectively, these bioinformatics data strongly suggest that fascin promotes proliferation through the SKP2-p27 axis.

**Figure 6:**
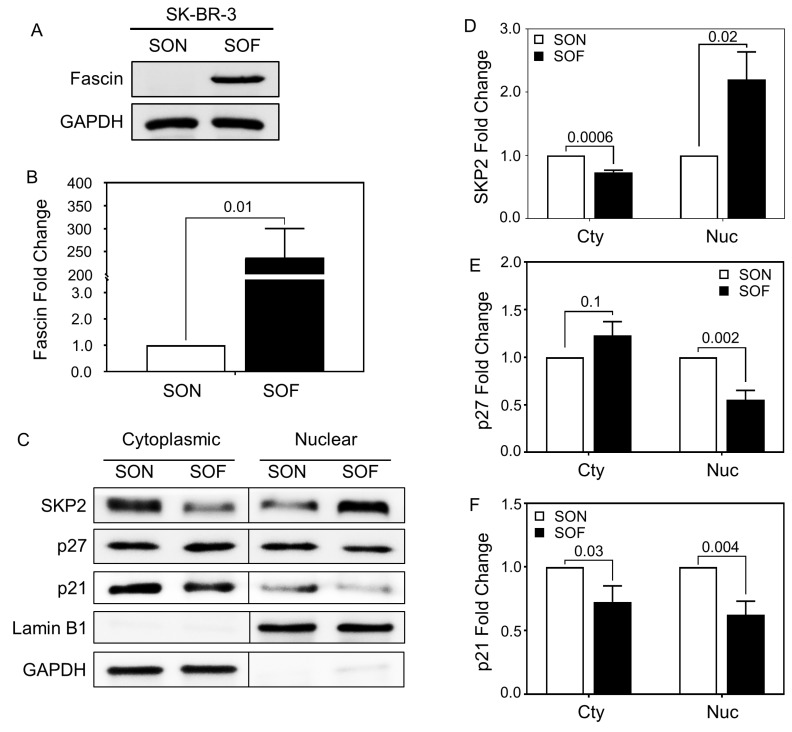
(**A**–**F**): Fascin induction in SK-BR-3 cells alters cell cycle checkpoint regulators. (**A**) Representative Western blot images showing total protein expression of fascin in SOF and SON. (**B**) Bar graph showing Western blot quantitation, and the results are the mean ± SEM of 3 independent experiments. (**C**) Representative Western blot images showing the expression of SKP2, p27, and p21 in the cytoplasmic (Cyt) and nuclear (Nuc) fraction of SOF and SON. Bar graph showing Western blot quantitation of SKP2 (**D**), p27 (**E**), and p21 (**F**), and the results are mean ± SEM of 3 independent experiments.

To functionally test the effect of fascin on the SKP2-p27/p21 axis, a Western blot was performed on the cytoplasmic and nuclear fractions of fascin-expressing cells (SOF) and their fascin-negative counterparts (SON). The nuclear fraction of SOF cells exhibited significantly higher expressions of SKP2 with reduced expressions of p27 and p21 compared to SON cells (Figure 6A–F). In the cytoplasmic fraction, SKP2 was significantly decreased in SOF, while p27 and p21 showed no significant differences between the two groups. Quantitative immunofluorescence analysis confirmed the Western blot findings, revealing elevated nuclear SKP2 and reduced nuclear p27 and p21 levels in SOF compared with their SON counterparts (Supplementary Figure S5). In MDA-MB-231 cells, the nuclear fraction of fascin knockdown (ShFSCN) showed significantly reduced SKP2 and increased p27 levels compared to their ShCon counterparts (Supplementary Figure S6A–E). Paradoxically, ShFSCN cells exhibited significantly reduced nuclear p21 expression (Supplementary Figure S6F), a pattern that differed from the observations in SK-BR-3 cells. The positive correlation of fascin with SKP2, coupled with its inverse association with p27 in two different BC subtypes, confirms the correlation observed in bioinformatics data and further supports fascin’s role in promoting proliferation through the modulation of this axis (SKP2 and p27) of the cell cycle checkpoint regulators.

### 3.5. Fascin Expression in BC Patient Tissues Correlates with Proliferation Markers and Poor Prognosis

**Figure 7:**
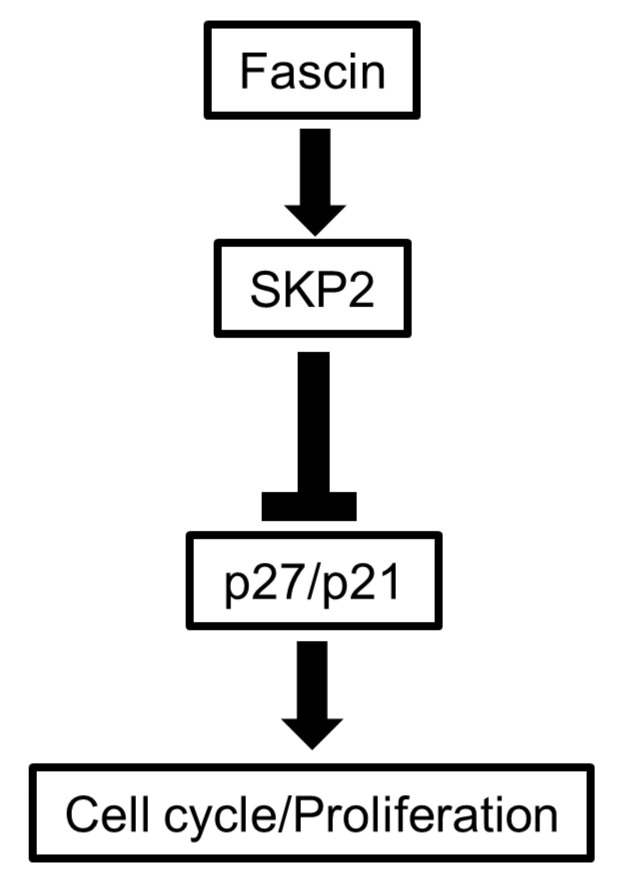
Schematic diagram illustrating that fascin drives cell cycle progression and promotes proliferation through upregulation of SKP2 and downregulation of p27/p21 expression.

To assess the relevance of bioinformatics and in vitro findings to patient samples, we leveraged immunohistochemistry to evaluate the relationship between fascin and the SKP2-p27/p21 axis in a cohort of 68 BC patient specimens (Supplementary Figure S7). While the original cohort included 71 cases [3], 3 cases were excluded from all protein staining due to tissue exhaustion. Fascin positivity was detected in 34% of the tested BC cases. Consistent with its association with higher proliferation, fascin expression significantly correlated with a higher histological grade (*p* = 0.0096) and Ki-67 (*p* = 0.0002) (Table 1). Fascin also exhibited a significant positive correlation with SKP2 (*p* = 0.0007) and an inverse relationship with p27 (*p* = 0.0208). No significant correlation (*p* = 0.2977) was observed with p21, though an inverse significant correlation with dual reduced p27/p21 was detected (*p* = 0.0298). Our data showed a significant correlation (*p* = 0.0157) between fascin and the immune checkpoint PD-L1, which we have recently reported to promote BC proliferation through the SKP2-p27/p21 axis [11]. Furthermore, fascin expression in our BC patients showed significant correlations with poor prognostic features, including the negative status of estrogen receptors (*p* ≤ 0.0001), progesterone receptors (*p* = 0.0009), and Her2/neu (*p* = 0.042). Altogether, these immunohistochemistry results confirm the bioinformatics and in vitro findings (Figure 7), highlighting fascin’s association with proliferation parameters and poor prognostic markers in BC patient samples.

## 4. Discussion

The oncogenic role of fascin in promoting cancer aggressiveness and poor clinical outcomes is well established. Although multiple studies have linked its expression with larger tumor sizes, whether fascin directly promotes cancer cell proliferation remains to be fully elucidated. This study demonstrates that fascin enhances BC cell proliferation by modulating key cell cycle checkpoint regulators that facilitate the transition from the G1 to S phase. These findings underscore fascin’s contribution to aggressive tumor behavior and its association with unfavorable clinical outcomes.

Although numerous studies described the oncogenic functions of fascin across various malignancies, its direct role in proliferation has been less frequently addressed. Schoumacher, M et al. generated a colon cancer mouse model using a tissue-specific conditional transgenic approach to induce fascin expression in the intestinal epithelium [14]. Fascin induction in this model reduced mice survival and accelerated tumor progression, indirectly suggesting a link between fascin and enhanced proliferation. Similarly, Ma et al. reported that fascin loss reduced both migration and proliferation of melanoblasts in vivo and melanoma cells in vitro, implicating fascin’s role in promoting cell growth [15]. In esophageal squamous cell carcinoma (ESCC), Xie et al. demonstrated through gain- and loss-of-function approaches that fascin regulates cell proliferation and invasiveness via the TGF-β pathway, which is also implicated in promoting epithelial-to-mesenchymal transition (EMT) in BC [7]. Consistently, Xing P et al. employed gain- and loss-of-function approaches in four different BC cell lines, showing that fascin expression promotes proliferation, enhances invasion potential, and enriches cancer stem cell-like features characterized by the CD44^+^/CD24^-^ and EMT phenotype [16]. Conversely, Heinz et al. showed that fascin induction enhances BC metastatic potential while exerting no effect on proliferation [17]. Collectively, these studies support a role for fascin in driving proliferation across multiple cancer types, including BC, although the underlying mechanisms remain incompletely defined.

Our study was driven by multiple observations linking fascin expression with proliferative activity. Bioinformatics data revealed a significant correlation between *FSCN1* expression and a proliferation signature score comprising 512 genes, including Ki-67 (*MKI67*), widely recognized as a global proliferation marker linked to rapid tumor growth and poor clinical outcomes as it is absent in quiescent cells (G0 phase) but present throughout the active phases of the cell cycle (G1, S, G2, and M) [18]. Consistently, the immunohistochemistry reflected this positive association between fascin and Ki-67 at the protein level. Fascin expression in BC tumor samples also showed a significant correlation with higher histological grade, which reflects tumor aggressiveness and often correlates with a higher mitotic index [19]. Importantly, in vitro gain- and loss-of-function experiments confirmed the bioinformatics and immunohistochemistry results and further demonstrated that fascin expression directly drives BC cell proliferation.

This study employed multiple approaches to explore the mechanisms underlying fascin-mediated BC proliferation. Bioinformatics analyses revealed a stronger correlation between *FSCN1* expression and both the G1-S signature score and the overall cell cycle signature score in *SKP2*^high^ tumors. While several correlations derived from bioinformatics analysis reached statistical significance (*p* < 0.001), the correlation coefficients were modest (r < 0.3), indicating that the strength of the association is weak despite being statistically significant. This likely reflects the large sample size of the METABRIC dataset and the multifactorial nature of gene expression in BC. Moreover, the mRNA data used in the bioinformatics analysis likely include transcripts from both malignant and stromal cells, making it difficult to attribute the correlations specifically to cancer cells. For this reason, we complemented these bioinformatics results with protein-level assessments specifically within cancer cells, using immunohistochemical analyses of patient-derived samples. Immunohistochemistry confirmed the association between fascin and SKP2, while in vitro experiments demonstrated that fascin promotes SKP2 upregulation in BC cells. SKP2 is known to drive cell cycle entry in quiescent cells by mediating the ubiquitination and degradation of p27 [20,21,22]. In BC cells, SKP2 expression has been shown to induce EMT and promote paclitaxel resistance [23]. In agreement, our previous work demonstrated that fascin also drives EMT and confers chemoresistance in BC cells [2,4], suggesting that fascin and SKP2 may act through a shared signaling pathway.

SKP2 promotes cell cycle progression by targeting the cyclin-dependent kinase inhibitors p27 and p21 for degradation, thereby enabling the G1-to-S-phase transition [24]. In many human cancers, including BC, reduced p27 expression has been linked to poor prognosis [25]. Consistent with our findings of fascin-mediated SKP2 upregulation, *FSCN1’s* correlation with the G1-S signature score was stronger in *CDKN1B*^low^ tumors, while *FSCN1’s* correlation with the cell cycle signature score was significant only in the *CDKN1B*^low^ cases. It is important to note that while bioinformatics data are based on mRNA, which may include signals from non-cancer cells, immunohistochemistry in patient samples and in vitro experiments specifically assess protein expression within BC cells. Thus, both immunohistochemistry and in vitro analyses independently confirm the correlation between fascin and the SKP2–p27 axis. Beyond transcriptional regulation, mutation in the C-terminal region of p27 has been reported across multiple malignancies, including BC. Of particular interest is the recurrent E171Stop mutation that generates a truncated protein (p271-170) in BC [26,27]. Future studies should explore the relationship between fascin expression in BC cells and p27 mutation. Finally, given that fascin expression correlates with PD-L1—as recently demonstrated by our group—to drive BC proliferation through the modulation of the SKP2-p27/p21 axis [11], we suggest that fascin promotes BC cell proliferation at least in part through the same axis.

The correlation between fascin and p21 was inconsistent across different models, likely due to multiple contributing factors, and thus this relationship should be interpreted cautiously. Bioinformatics analyses unexpectedly revealed a statistically significant positive, albeit weak, correlation between fascin and p21, which may reflect the post-transcriptional regulation of p21 at the protein level. Previous studies have demonstrated that the PI3K/AKT and SKP2 pathways can regulate p21 protein expression through both transcriptional and post-transcriptional mechanisms [28,29,30]. At the protein level, an inverse correlation between fascin and p21 was observed in SK-BR-3 cells; however, this relationship was not detected in MDA-MB-231 cells. This discrepancy may reflect intrinsic biological differences between BC subtypes, as SK-BR-3 and MDA-MB-231 represent HER2-positive and triple-negative BC models, respectively. Moreover, although SKP2 post-transcriptionally regulates both p27 and p21, each can also be modulated by distinct signaling pathways. For example, p21 is controlled by both p53-dependent (noting that p53 is mutated in MDA-MB-231) and p53-independent mechanisms [31], while p27 is influenced by growth factor signaling [32]. Consistent with these complex regulatory networks, the immunohistochemical analysis of patient samples revealed no significant inverse associations between fascin and p21 alone. However, BC patients with the concurrent loss of both p27 and p21 showed a significant correlation with SKP2 (*p* = 0.004) and fascin (*p* = 0.0396) expression.

Fascin may regulate the SKP2–p27 axis through multiple mechanisms. Our previous studies showed that fascin confers drug resistance in BC cells through the activation of the focal adhesion kinase (FAK)-PI3K/Akt signaling pathway [2]. Similarly, fascin was shown to promote the proliferation of human hepatic satellite cells through the FAK-PI3K/Akt signaling axis [33]. Given this observation and prior evidence that PI3K/Akt signaling upregulates SKP2 transcription in pancreatic ductal adenocarcinoma cells [34], it is plausible that fascin enhances SKP2 expression through PI3K/Akt activation. Huang et al. identified SKP2 as a direct transcriptional target of STAT3 and IL-6 activation in human cervical carcinoma cells [35], whereas Snyder et al. showed that IL-6 and TNF-α regulate STAT3 and NF-κB binding to the promoter region of fascin and induce its expression in BC cells [36]. We further reported that fascin itself activates NF-κB in BC cells following TNF-α stimulation [3], suggesting that overlapping signaling pathways may coordinately regulate fascin and SKP2 expression. A limitation of this study is the lack of direct assessments of whether the fascin-mediated effects on cell cycle progression are SKP2-dependent. Future studies should investigate this phenomenon by utilizing an SKP2-knockdown approach in the presence or absence of fascin. Finally, the inconsistent correlation observed between fascin and p21 across different tested models underscores the need for future studies, particularly in larger patient cohorts and in specific molecular BC subtypes.

## 5. Conclusions

This study establishes a direct role for fascin in promoting BC cell proliferation through the modulation of the SKP2–p27 signaling axis. It integrates computational, in vitro, and in vivo evidence to demonstrate that fascin promotes BC cell cycle progression—at least in part—by upregulating SKP2 and downregulating its inhibitory targets, p27 and p21, thereby facilitating the G1-to-S-phase transition. Together, these findings reveal a previously underappreciated oncogenic role of fascin and highlight its promise as a potential therapeutic target to suppress tumor aggressiveness and improve clinical outcomes in BC.

## Figures and Tables

**Table 1:** Correlation between fascin and clinicopathological parameters as well as markers of proliferation in 68 breast cancer patients.

		Fascin		
		−	+	* *p*
**CP**	**Age**			
	<40 years	15 ^§^ (71)	6 (29)	0.591
	≥40 years	30 (64)	17 (36)	
			
	**Histological Grade**			
	I&II	28 (82)	6 (18)	**0.0096**
	III	17 (50)	17 (50)	
**BC Subtype**	**ER Status**			
	Negative	7 (29)	17 (71)	**<0.0001**
	Positive	38 (86)	6 (14)	
			
	**PR Status**			
	Negative	18 (49)	19 (51)	**0.0009**
	Positive	27 (87)	4 (13)	
			
	**Her2/neu Status**			
	Negative	31 (70)	13 (30)	**0.04219**
	Positive	14 (58)	10 (42)	
**IE**	**PD-L1**			
	<5%	41 (37)	15 (27)	**0.0157**
	≥5%	4 (33)	8 (67)	
**Proliferation**	**Ki-67**			
	<20%	34 (85)	6 (15)	**0.0002**
	≥20%	11 (39)	17 (61)	
			
	**SKP2**			
	<10%	37 (80)	9 (20)	**0.0007**
	≥10%	8 (36)	14 (64)	
			
	**Loss of p27**			
	<50%	29 (58)	21 (42)	**0.0208**
	≥50%	16 (89)	2 (11)	
			
	**Loss of p21**			
	<10%	24 (60)	16 (40)	0.2977
	≥10%	21 (75)	7 (25)	
			
	**Loss of (p27/p21)**			
	No	15 (52)	14 (48)	**0.0396**
	Yes	30 (77)	9 (23)	

Abbreviations: + and − are the number of positive and negative patients. ^§^ Numbers between brackets are the percentages of patients. * *p* values in bold represent statistically significant data. Clinicopathological (CP) and immune evasion (IE) markers. Loss refers to downregulation.

## Data Availability

The data generated in this current study are included in this published manuscript (and its Supplementary Materials); otherwise, data are available from the corresponding author upon reasonable request.

## Supplementary Materials

The following supporting information can be downloaded at: https://www.mdpi.com/article/10.3390/cells14231839/s1, Supplementary Figure S1. (A,B): Fascin induction in SK-BR-3 BC cell line. Fascin ORF or empty ORF were used to generate fascin expressing (SOF) or control (SON) SK-BR-3 cells. Fascin ORF or empty ORF were used to generate fascin expressing (SOF) or control (SON) SK-BR-3 cells. (A) Bar graph of RT-qPCR showing relative expression of *FSCN1* mRNA in SK-BR-3 cells. The results (mean ± SD) is representative of 3 independent experiments. (B) Representative flow cytometry histograms showing fascin protein expression in SK-BR-3 cells. Numbers on histograms are the percentage of fascin positive cells as determined in reference to the isotype control. Supplementary Figure S2. (A,B): Fascin d knockdown in MDA-MB-231 BC cell line. Scrambled or fascin ShRNA were used to generate fascin expressing (ShCon) or knockdown (ShFSCN) MDA-MB-231 cells. Scrambled or fascin ShRNA were used to generate fascin expressing (ShCon) or knockdown (ShFSCN) MDA-MB-231 cells. (A) Bar graph of RT-qPCR showing relative expression of *FSCN1* mRNA in MDA-MB-231 cells. The results (mean ± SD) is representative of 3 independent experiments. (B) Representative flow cytometry histograms showing fascin protein expression in MDA-MB-231 cells. Numbers on histograms are the percentage of fascin positive cells as determined in reference to the isotype control. Supplementary Figure S3: Fascin expression in BC patients correlates with SKP2. Scatter plots showing correlation between the expression of *FSCN1* and *SKP2* using METABRIC BC dataset (*n* = 1980). Pearson’s correlation coefficient (R) and *p*-value are displayed on the plot. Supplementary Figure S4. (A–F): Fascin correlation with the cell cycle signature score is SKP2-dependent. Scatter plot showing the correlation between *FSCN1* expression and 1705 cell cycle gene signature score (A) and (D) using the METABRIC BC dataset (*n* = 1980). Patients were stratified into (B) *SKP2*^high^ (n = 990) and (C) *SKP2*^low^ (*n* = 990) groups based on the median *SKP2* expression level. Patients were stratified into (E) *CDKN1B*^high^ (*n* = 990) and (F) *CDKN1B*^low^ (*n* = 990) groups based on the median *CDKN1B* expression level. Scatter plots illustrate the correlation between fascin expression and the G1-S signature score within each subgroup. Pearson’s correlation coefficients (R) and corresponding *p*-values are displayed on each plot. Supplementary Figure S5. (A,B): Fascin expression in SK-BR-3 cells increased SKP2 and reduced p27. (A) Representative images of SKP2 (top), p27 (middle) and p21 (bottom) in SON (left) and SOF (right) at 200x magnification. B) Bar graph showing the expression levels of SKP2 (top), p27 (middle) and p21 (bottom) in SOF after normalization to the SON as measure by quantitative immunefluorescence and the results are displayed as mean ± SEM. Supplementary Figure S6. (A–F): Fascin knockdown in MDA-MB-231 cells modifies the cell cycle checkpoint regulators. (A) Representative western blot images showing total protein expression of fascin in ShCon and ShFSCN. (B) Bar graph showing western blot quantitation and the results are mean ± SEM of 3 independent experiments. (C) Representative western blot images showing the expression of SKP2, p27, and p21 in the cytoplasmic (Cyt) and nuclear (Nuc) fraction of ShCon and ShFSCN. Bar graph showing western blot quantitation of SKP2 (D), p27 (E), p21 (F) and the results are mean ± SEM of 3 independent experiments. Supplementary Figure S7: Representative immunohistochemical images showing the expression of SKP2, p27, and p21 in breast cancer patients (x400 magnification).

## Abbreviations

The following abbreviations are used in this manuscript:

BC	Breast Cancer
EMT	Epithelial-to-Mesenchymal Transition
FAK	Focal Adhesion Kinase
