# Supplementary material for: Fascin Drives Breast Cancer Cell Proliferation Partly by Modulating the Cell Cycle Checkpoint Regulators of the G1-S Phase
Source: Cells. 2025 Nov 21;14(23):1839. doi: 10.3390/cells14231839 (PMC12690981; doi:10.3390/cells14231839)
Supplement: Supplementary file 1 [file cells-14-01839-s001.zip › cells-3945121-supplementary.pdf]

Supplementary Figure S1. (A,B)

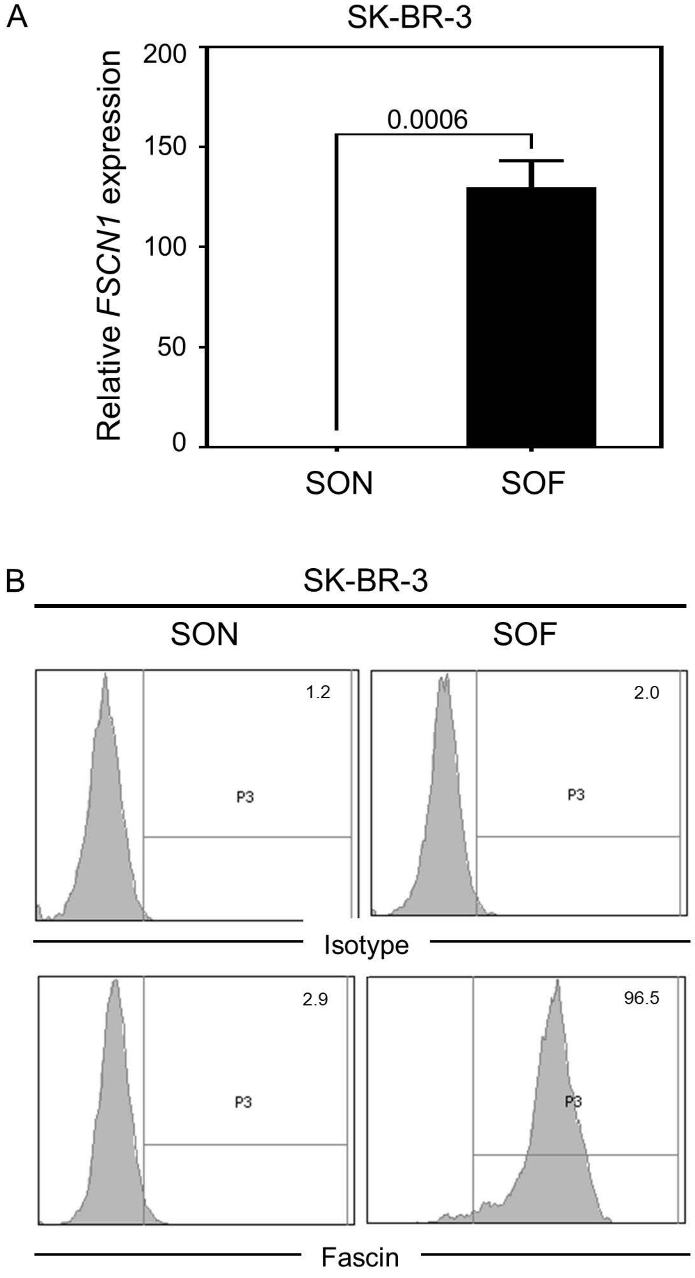

Supplementary Figure S1 (A,B): Fascin induction in SK-BR-3 BC cell line. Fascin ORF or empty ORF were used to generate fascin expressing (SOF) or control (SON) SK-BR-3 cells. A) Bar graph of RT-qPCR showing relative expression of *FSCN1* mRNA in SK-BR-3 cells. The results (mean ± SD) is representative of 3 independent experiments. B) Representative flow cytometry histograms showing fascin protein expression in SK-BR-3 cells. Numbers on histograms are the percentage of fascin positive cells as determined in reference to the isotype control.

Supplementary Figure S2. (A,B)

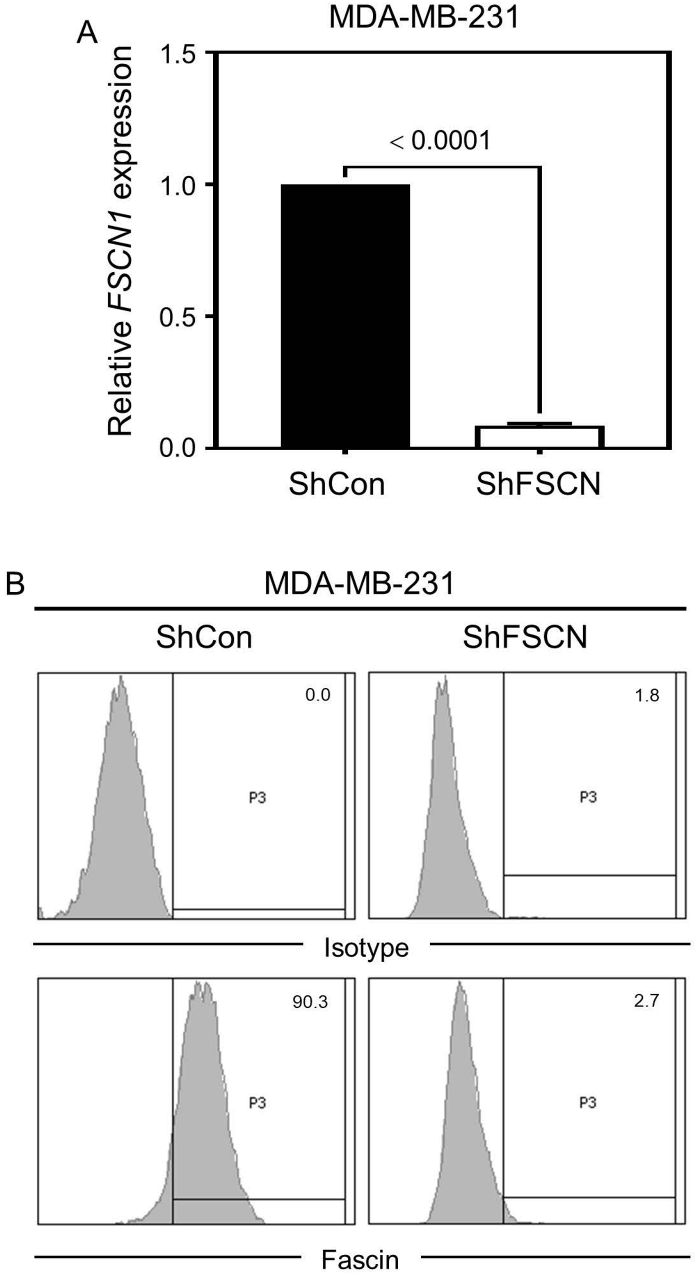

Supplementary Figure S2 (A,B): Fascin d knockdown in MDA-MB-231 BC cell line. Scrambled or fascin ShRNA were used to generate fascin expressing (ShCon) or knockdown (ShFSCN) MDA-MB-231 cells. A) Bar graph of RT-qPCR showing relative expression of *FSCN1* mRNA in MDA-MB-231 cells. The results (mean ± SD) is representative of 3 independent experiments. B) Representative flow cytometry histograms showing fascin protein expression in MDA-MB-231 cells. Numbers on histograms are the percentage of fascin positive cells as determined in reference to the isotype control.

Supplementary Figure S3.

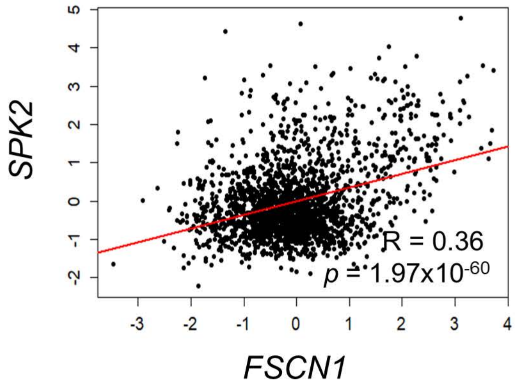

Supplementary Figure S3: Fascin expression in BC patients correlates with SKP2. Scatter plots showing correlation between the expression of *FSCN1* and *SKP2* using METABRIC BC dataset (n = 1980). Pearson's correlation coefficient (r) and *p*-value are displayed on the plot.

Supplementary Figure S4. (A–F)

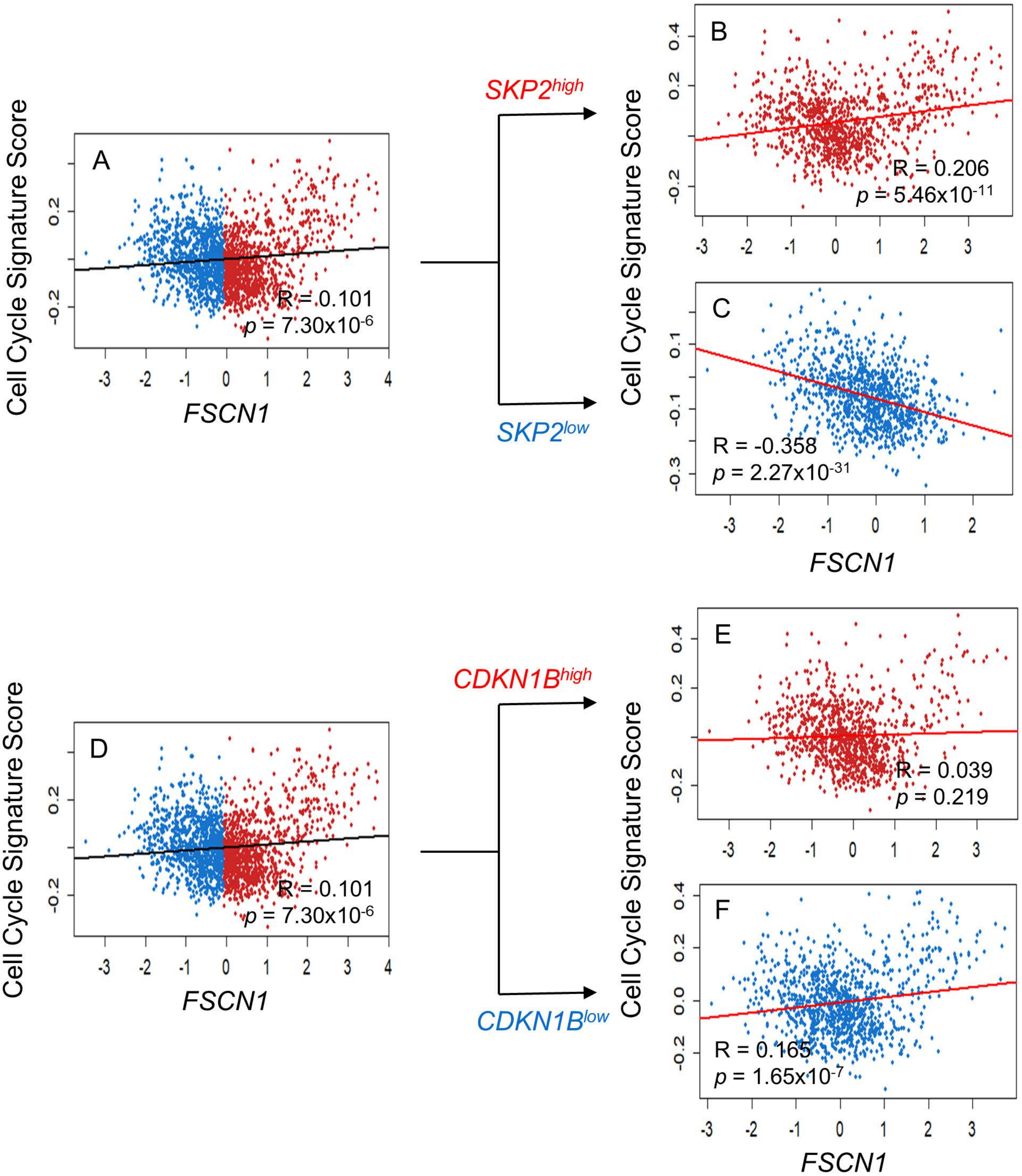

Supplementary Figure S4. (A–F): Fascin correlation with the cell cycle signature score is SKP2-dependent. Scatter plot showing the correlation between *FSCN1* expression and 1705 cell cycle gene signature score (A) and (D) using the METABRIC BC dataset (n = 1980). Patients were stratified into (B) *SKP2*^{high} (n = 990) and (C) *SKP2*^{low} (n = 990) groups based on the median *SKP2* expression level. Patients were stratified into (E) *CDKN1B*^{high} (n = 990) and (F) *CDKN1B*^{low} (n = 990) groups based on the median *CDKN1B* expression level. Scatter plots illustrate the correlation between fascin expression and the G1-S signature score within each subgroup. Pearson's correlation coefficients (R) and corresponding p-values are displayed on each plot.

Supplementary Figure S5. (A,B)

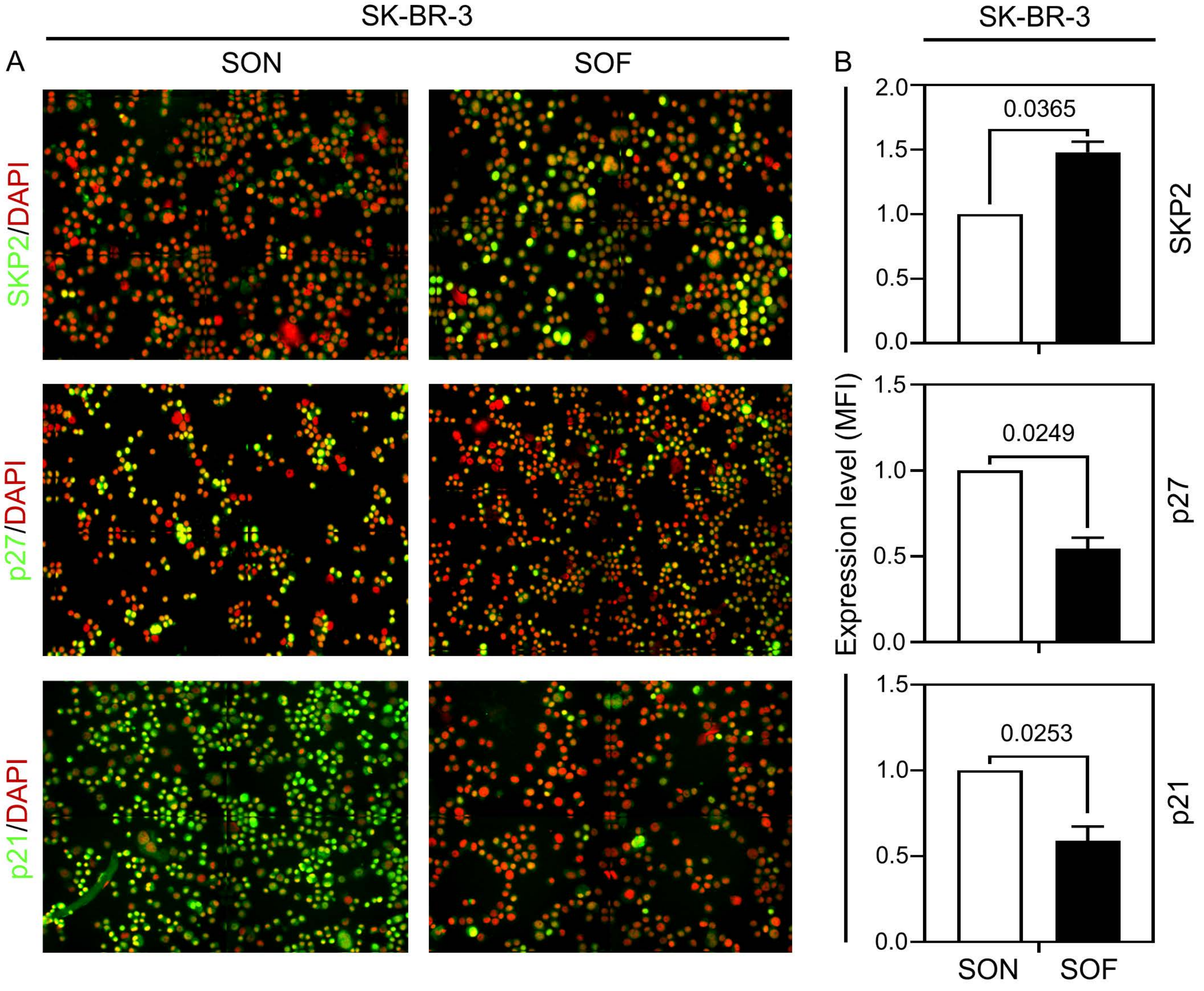

Supplementary Figure S5. (A,B): Fascin expression in SK-BR-3 cells increased SKP2 and reduced p27. A) Representative images of SKP2 (top), p27 (middle) and p21 (bottom) in SON (left) and SOF (right) at 200x magnification. B) Bar graph showing the expression levels of SKP2 (top), p27 (middle) and p21 (bottom) in SOF after normalization to the SON as measure by quantitative immunefluorescence and the results are displayed as mean ± SEM.

Supplementary Figure S6. (A–F)

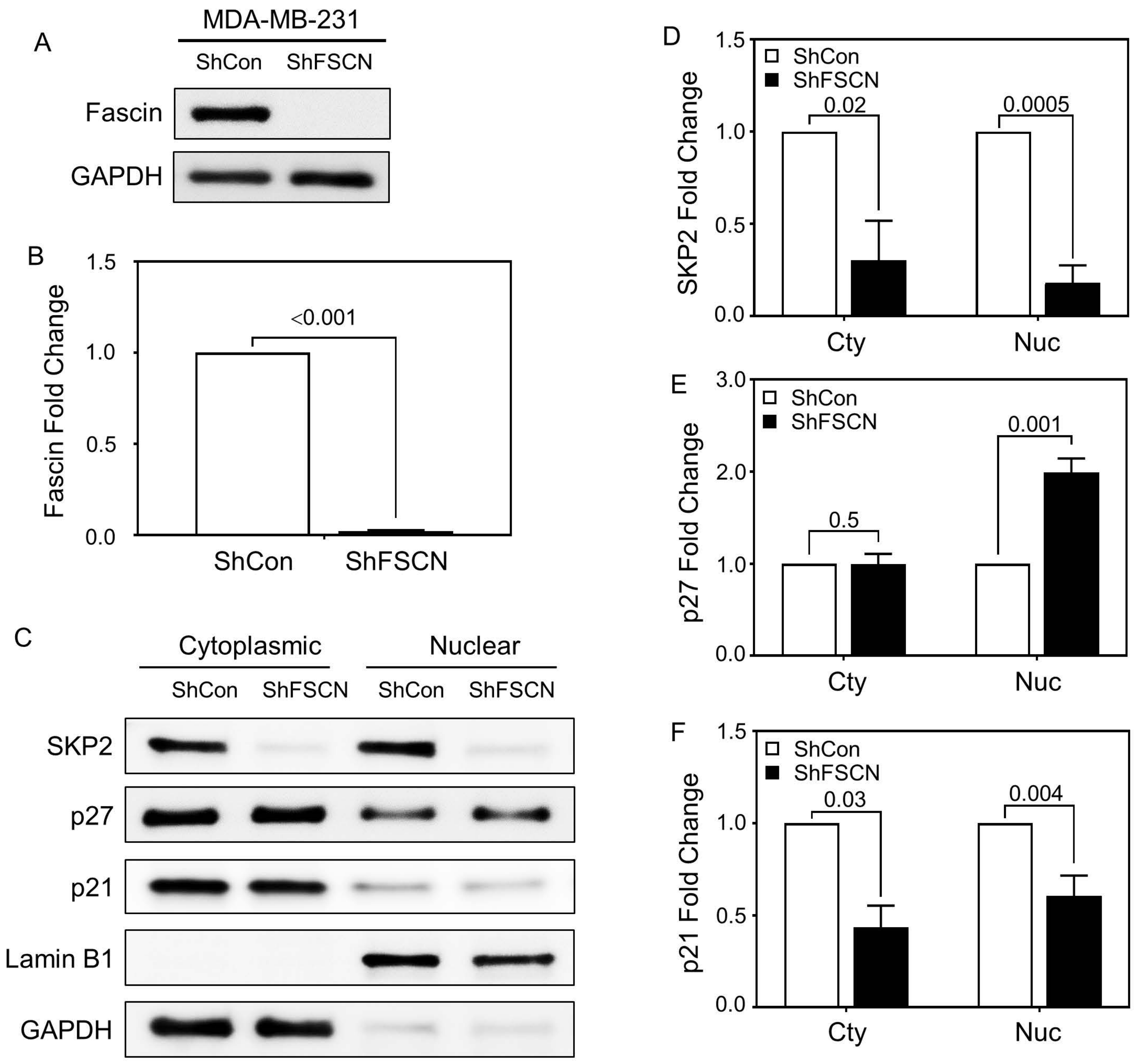

Supplementary Figure S6. (A–F): Fascin knockdown in MDA-MB-231 cells modifies the cell cycle checkpoint regulators. A) Representative western blot images showing total protein expression of fascin in ShCon and ShFSCN. B) Bar graph showing western blot quantitation and the results are mean ± SEM of 3 independent experiments. C) Representative western blot images showing the expression of SKP2, p27, and p21 in the cytoplasmic (Cyt) and nuclear (Nuc) fraction of ShCon and ShFSCN. Bar graph showing western blot quantitation of SKP2 (D), p27 E), p21 (F) and the results are mean ± SEM of 3 independent experiments.

Supplementary Figure S7.

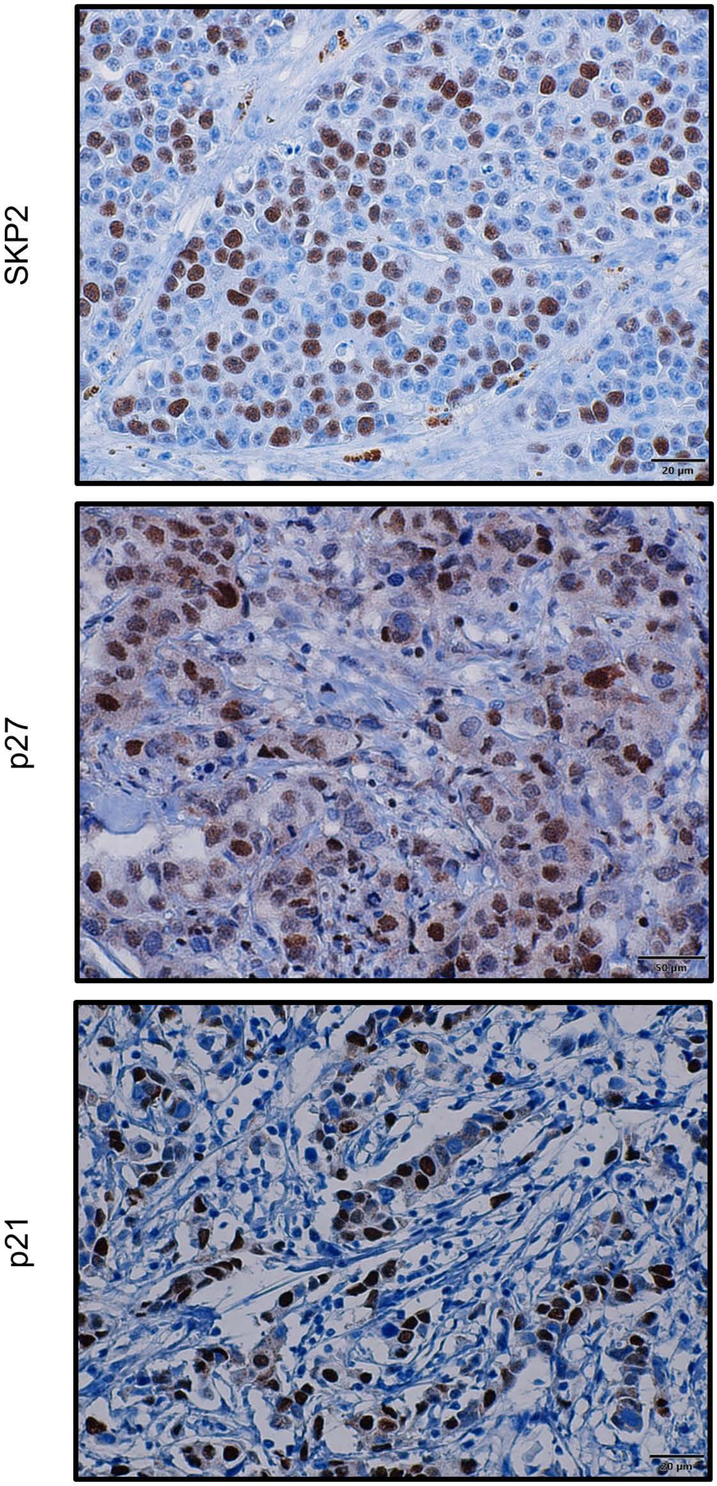

Supplementary Figure S7: Representative immunohistochemical images showing the expression of SKP2, p27, and p21 in breast cancer patients (x400 magnification).
